# Decoding microbial carcinogenic strategies: ubiquitination and SUMO modification

**DOI:** 10.3389/fmicb.2025.1720153

**Published:** 2025-12-19

**Authors:** Yue Liu, Xianghai Zeng, Zhimai Lyu, Dandan Huang

**Affiliations:** 1 School of Basic Medicine, Gannan Medical University, Ganzhou, Jiangxi, China; 2 Department of Neurology, First Affiliated Hospital of Gannan Medical University, Ganzhou, Jiangxi, China

**Keywords:** carcinogenic microorganisms, infection-associated carcinogenesis, targeted therapies, tumorigenesis mechanisms, ubiquitin-SUMO axis

## Abstract

Carcinogenic microorganisms (including viruses, bacteria, fungi, etc.) disrupt cellular homeostasis to drive tumorigenesis by hijacking the host ubiquitin-proteasome system (UPS) and SUMOylation networks, with oncogenic viruses representing the core agents of this regulatory mechanism. Specifically: - Human papillomavirus (HPV) E6 protein binds E3 ubiquitin ligase E6AP to mediate ubiquitin-mediated degradation of tumor suppressor p53, thereby disabling cell cycle surveillance; the HBx protein of hepatitis B virus (HBV) evades its own ubiquitin-mediated degradation by inhibiting the activity of the E3 ligase SIAH1, while simultaneously upregulating DNA methyltransferases to disrupt host epigenetics; the core protein of hepatitis C virus (HCV) induces methylation of the E6AP promoter, blocking its own ubiquitin-mediated degradation to maintain oncogenic activity; Epstein–Barr virus (EBV) LMP1 activates IRF7 via K63-linked ubiquitination, sustaining NF-xB pathway activation to promote proliferation; Kaposi’s sarcoma-associated herpesvirus (KSHV) K3 protein mediates MHC-I molecule ubiquitination-dependent endocytosis, achieving immune evasion. Furthermore, non-viral microorganisms such as *Helicobacter pylori* CagA and aflatoxin A also participate in carcinogenesis by regulating the UPS/SUMO system. In summary, targeted modulation of the UPS/SUMO system constitutes a core oncogenic strategy for carcinogenic microorganisms (particularly viruses), providing molecular targets for precision cancer therapy.

## 1 Introduction

Tumorigenesis is a complex multifactorial process involving the synergistic effects of genetic predisposition, environmental exposure, lifestyle factors, and microbial infections. In recent years, the role of microorganisms in carcinogenesis has garnered increasing attention. The World Health Organization (WHO) has explicitly classified multiple microorganisms as Group 1 carcinogens, including *Helicobacter pylori* (*Hp*), Epstein–Barr virus (EBV), high-risk human papillomavirus (HPV), hepatitis B virus (HBV), and hepatitis C virus (HCV) (National Toxicology Program, 2021). Notably, these pathogens tend to establish chronic persistent infections—long-term colonization through immune evasion strategies. This not only directly induces tissue damage (e.g., chronic inflammation and fibrosis) but may also drive cellular malignant transformation via mechanisms like genomic integration. Their carcinogenic mechanisms exhibit high diversity, encompassing genotoxic damage, epigenetic reprogramming, abnormal activation of oncogenic signaling pathways, persistent inflammatory responses, dysregulated immune responses, and metabolic pathway remodeling (Wizenty and Sigal, 2025; Chen and Zhao, 2025; Zhao H. J. et al., 2022; Péneau et al., 2022; Li and Gao, 2021).

Thanks to rapid advances in metagenomics, proteomics, and related technologies, the molecular networks through which microbes regulate host cells are becoming increasingly clear. Studies indicate that various microbial effector molecules can specifically manipulate host post-translational modification (PTM) systems, with the ubiquitination and SUMOylation pathways receiving particular attention as key regulatory hubs (Zhu et al., 2025; Mukherjee and Dikic, 2022). By reprogramming these two modification networks within host cells, microorganisms precisely regulate the stability, subcellular localization, and functional interactions of key proteins (including microbial proteins, host tumor suppressors, and oncoproteins), ultimately creating a favorable intracellular environment for tumorigenesis and progression (Li et al., 2025; You et al., 2023).

**Figure 1:**
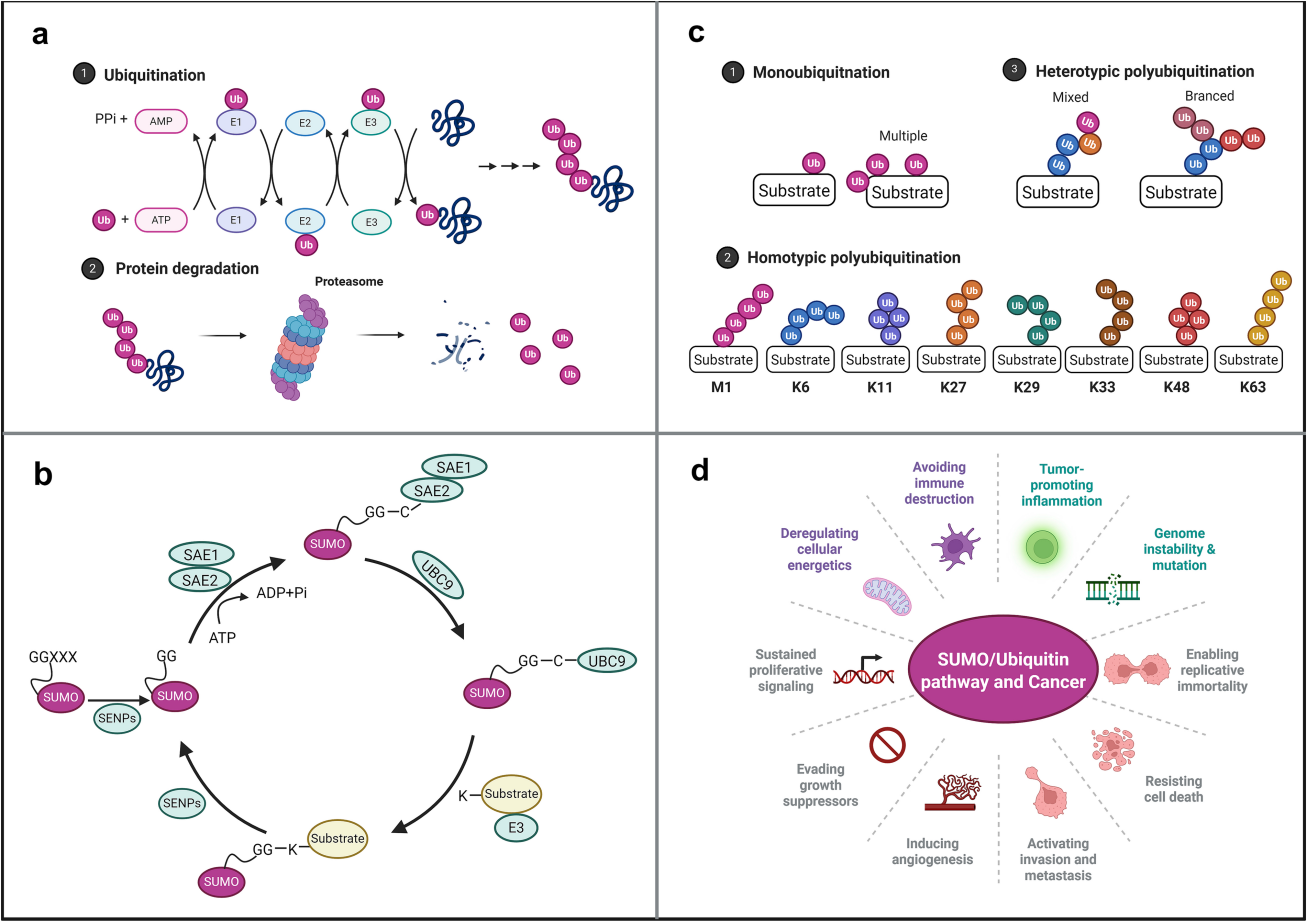
Ubiquitination and SUMOylation: Modification regulation of cell survival and proliferation in cancer **(a)** ubiquitination process (Sampson et al., 2023). **(b)** SUMOylation process (Vertegaal, 2022). **(c)** Chain types in ubiquitination (Xu et al., 2009). **(d)** Role of ubiquitination/SUMOylation pathways in cancer (Han et al., 2018; Liu F. et al., 2024).

At the molecular mechanism level, both the ubiquitination and SUMOylation modification systems rely on ATP-driven E1–E2–E3 enzyme cascade reactions to covalently attach ubiquitin or SUMO molecules to lysine residues of substrate proteins. However, they exhibit differences in catalytic systems, chain topological structures, and biological functions (Zheng and Shabek, 2017; Müller et al., 2001). In regulating cell survival and proliferation, the ubiquitin pathway primarily employs K48-linked polyubiquitin chains to mark substrate proteins (e.g., the tumor suppressor p53), directing their degradation by the proteasome and directly driving tumorigenesis (Xia et al., 2024). Additionally, non-degradative polyubiquitin chain modifications such as K63 are extensively involved in crucial physiological processes including DNA damage repair, NF-κB pathway activation, and cell cycle progression (Liu et al., 2018; Martínez-Férriz et al., 2022). SUMOylation, conversely, functions as a dynamic switch regulating protein activity. Known SUMO proteins—SUMO1, SUMO2/3, SUMO4, and SUMO5—modify transcription factors (p53, NF-κB, etc.) to regulate their activity, thereby influencing DNA repair, cell cycle progression, and genomic stability. This constitutes a core mechanism governing cellular fate (Figure 1) (Han et al., 2018).

This review aims to systematically elucidate the molecular mechanisms by which microbial hijacking of host ubiquitination and SUMOylation systems drives tumorigenesis, while exploring novel therapeutic strategies targeting this axis to establish a theoretical foundation for developing precision intervention approaches.

## 2 Elucidating mechanisms by which oncogenic microorganisms manipulate ubiquitination and SUMOylation pathways to drive tumorigenesis

### 2.1 HPV

HPV is the most common sexually transmitted viral infection affecting both women and men worldwide. Epidemiological data indicate that 85% of women and 95% of sexually active men acquire HPV infection at some point in their lives (Chesson et al., 2014; Sabeena et al., 2017). Nearly all cervical cancers, most anal cancers, and a significant proportion of non-cervical malignancies (e.g., vaginal, penile, vulvar, and oropharyngeal cancers) have been confirmed to be closely associated with HPV infection (Jain et al., 2023). In recent years, a growing body of research indicates that HPV oncoproteins can manipulate the ubiquitin system to drive cell proliferation and prevent apoptosis, ultimately promoting cellular transformation (Wootton and Morgan, 2025).

**Figure 2:**
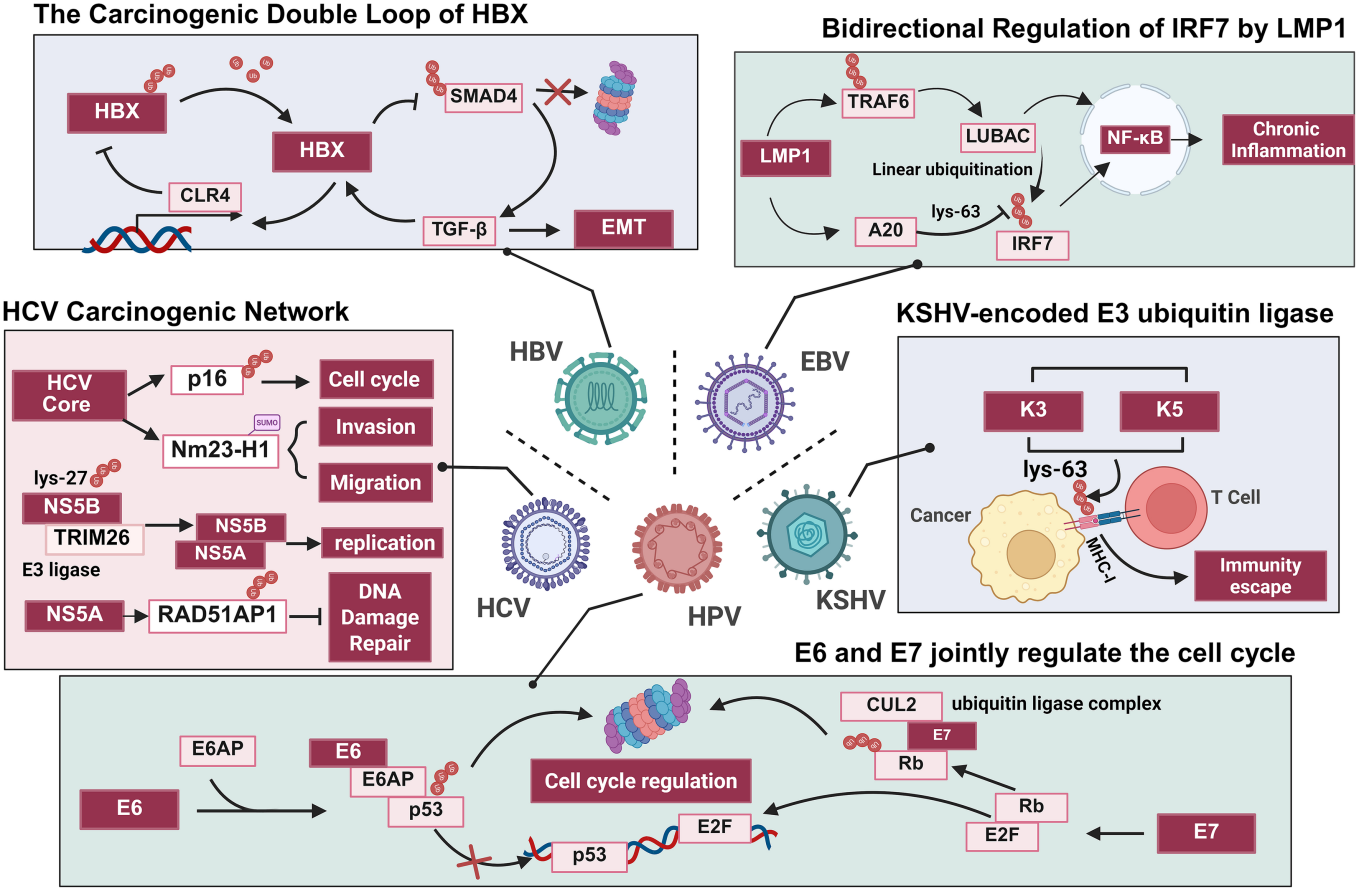
The crucial mechanism by which key proteins encoded by five major oncogenic viruses promote tumorigenesis through ubiquitination and SUMOylation modifications. HPV disrupts cell cycle regulation via its E6 and E7 proteins (Scheffner et al., 1993; Huh et al., 2007), HBV regulates TGF-β expression via a biphasic mechanism mediated by the HBX protein (Chaomin et al., 2022; Xie et al., 2025); HCV participates in tumorigenesis through multiple pathways (Nguyen et al., 2018; Cha et al., 2021; Paul et al., 2019; Liang et al., 2021); EBV’s LMP1 protein regulates IRF7 via ubiquitination, thereby activating the NF-κB pathway (Wang et al., 2017; Ning and Pagano, 2010); KSHV encodes a viral E3 ubiquitin ligase that mediates MHC-I ubiquitination, facilitating immune evasion (Ning and Pagano, 2010).

Delving into its carcinogenic mechanisms, the roles of HPV oncoproteins E6 and E7 are particularly pivotal. HPV E6, through the presence of an LXXLL binding motif, forms a stable heterotrimeric complex comprising E6, E3 ubiquitin ligase E6AP, and p53. This complex stimulates E6AP E3 ligase activity, promoting p53 polyubiquitination and proteasomal degradation (Figure 2) (Scheffner et al., 1993; Huibregtse et al., 1993; Martinez-Zapien et al., 2016). p53 degradation leads to uncontrolled cell cycle progression, impaired apoptosis, and contributes to tumor immune evasion (Liu et al., 2023). Interestingly, while low-risk HPV E6 can also bind E6AP, it does not target p53 (Brimer et al., 2007). Beyond the classic p53 pathway, the E6-E6AP complex targets numerous other proteins. Recent studies identified approximately 190 potential substrate proteins for HPV-11 E6 and HPV-16 E6 (Ebner et al., 2020). Additionally, E3 ubiquitin ligase UBR5 and MARCHF8 are downstream molecules of E6 protein, playing crucial roles in tumorigenesis (Subbaiah et al., 2016; Khalil et al., 2023). Evidence indicates that HPV E6 induces the degradation of the deubiquitinating enzyme CYLD, thereby activating the NF-κB signaling pathway and promoting cervical cancer cell proliferation (HeLa, SiHa, and Me180) (An et al., 2008; Tilborghs et al., 2017). Concurrently, E6 forms a complex with the deubiquitinating enzyme USP46 in cervical cancer cells, promoting interaction between USP46 and Cdt2 (a component of the CRL4^Cdt2^ E3 ubiquitin ligase complex). This interaction maintains the stability of Cdt2 and, thereby driving cell proliferation (HeLa and U2OS) (Kiran et al., 2018; Kiran et al., 2021).

HPV16 E7 primarily mediates the ubiquitination and degradation of retinoblastoma protein (pRB) through interactions with the E3 ubiquitin ligase complex (comprising ZYG11B/ZER1, CUL2, Rbx1, Elongin B, and C) (Figure 2) (Huh et al., 2007; White et al., 2012; Fischer et al., 2017; Nouel and White, 2022). The degradation of pRB releases E2F transcription factors, activating transcription and driving cell cycle progression (Mandigo et al., 2022). Furthermore, HPV E7 recruits the E3 ubiquitin ligase TRIM21, ubiquitinating and degrading the inflammasome IFI16, thereby inhibiting pyroptosis and aiding viral escape from immune surveillance (HeLa and 293 T) (Song et al., 2020). Additionally, E7 has been found to bind to the E3 ubiquitin ligase UBR4, targeting the protein tyrosine phosphatase PTPN14 for proteasomal degradation (Huh et al., 2005; White et al., 2016; Szalmás et al., 2017). PTPN14 acts as a negative regulator of the oncogenic transcription factor YAP1 and can activate the upstream Hippo signaling pathway by interacting with large tumor suppressor kinase 1 (LATS1) (Liu et al., 2013; Wilson et al., 2014). Given the importance of YAP/TAZ and Hippo signaling in HPV + cancers, E7-mediated PTPN14 degradation may represent a key mechanism for E7-driven tumorigenesis (He et al., 2015; Hatterschide et al., 2019; Patterson et al., 2024). It is important to emphasize that the expression levels of HPV E6 and E7 themselves are also subject to complex and precise regulation by the ubiquitin-proteasome system (Wootton and Morgan, 2025). Collectively, these findings highlight the central role of ubiquitin modification in HPV-driven cancer development.

Beyond the ubiquitin pathway, HPV oncoproteins also drive tumorigenesis by interfering with SUMOylation. A key mechanism involves high-risk HPV E6 protein targeting and degrading the SUMO ligase UBC9 (Heaton et al., 2011). This action causes a widespread reduction in overall SUMOylation levels within host cells, disrupting the normal differentiation program of keratinocytes. This maintains them in an undifferentiated proliferative state, creating a favorable environment for viral replication and carcinogenesis (293A, C33A, and HeLa) (Heaton et al., 2011). Notably, E6’s regulation of the SUMOylation network is complex and multifaceted. It not only globally suppresses SUMOylation by degrading UBC9 but also specifically enhances SUMOylation modifications of certain key host proteins. For example, studies confirm that E6 promotes SUMOylation of the transcription coactivator hADA3, and this modification enhances hADA3 degradation via the ubiquitin-proteasome pathway (Chand et al., 2014). As a crucial regulator of the p53 pathway, degradation of hADA3 significantly weakens cellular tumor suppression, thereby promoting proliferation and inhibiting apoptosis (Chand et al., 2014; Wang et al., 2001). Furthermore, the upregulation of UBC9 itself has been implicated in the progression of head and neck cancer (HNC). It induces epithelial-mesenchymal transition (EMT) by downregulating E-cadherin, thereby promoting tumor invasion (Wang et al., 2001). Similarly, UBC9 expression levels have been identified as a potential diagnostic biomarker for cervical cancer (Mattoscio et al., 2015). Therefore, delving into the molecular mechanisms underlying the interaction between HPV oncoproteins and SUMOylation, and elucidating how abnormal expression or dysfunction of SUMOylation specifically drives the development of HPV-associated cancers—particularly its regulation of key carcinogenic processes such as EMT—is crucial for comprehensively understanding HPV carcinogenesis and developing novel therapeutic intervention strategies (Table 1).

**Table 1:** Mechanism of HPV oncoprotein carcinogenesis via the ubiquitination and SUMOylation.

Viral protein	UPS/SUMO activity	Target	Main mechanism	References
HPV E6	E6-AP (E3 ligase)	p53	Degradation of p53, disrupting cell-cycle control, inhibiting apoptosis, and contributing to tumor immune evasion	Scheffner et al. (1993), Huibregtse et al. (1993), Martinez-Zapien et al. (2016), and Liu et al. (2023)
	CYLD (deubiquitinase)	NF-κB (K63)	Activating NF-κB signaling and enhancing cervical cancer cell proliferation	An et al. (2008) and Tilborghs et al. (2017)
	USP46 (deubiquitinase)	Cdt2	Facilitating USP46–Cdt2 interaction, stabilizing Cdt2, thus promoting proliferation	Kiran et al. (2018) and Kiran et al. (2021)
	Ubc9 (E2 ligase)	hADA3	Degradation of p53, weakening the tumor suppressor ability of cells, thereby promoting proliferation and inhibiting apoptosis	Chand et al. (2014) and Wang et al. (2001)
	Ubc9 (E2 ligase)		Promoting EMT via downregulation of E-cadherin, leading more aggressive tumor phenotype	Wang et al. (2001)
HPV E7	Cullin 2 ubiquitin ligase complex (E3 ligase)	pRB	Inducing ubiquitin-mediated degradation of pRB, activating transcription and drive the progress of the cell cycle	Huh et al. (2007), White et al. (2012), Fischer et al. (2017), and Nouel and White (2022)
	TRIM21 (E3 ligase)	IFI16 (K33)	Degradation of IFI16, suppressing pyroptosis and facilitating viral immune evasion	Song et al. (2020)
	UBR4 (E3 ligase)	PTPN14	Degradation of PTPN14 and activation of YAP1 leading to activation of the pro-oncogenic pathway	Huh et al. (2005), White et al. (2016), Szalmás et al. (2017), Liu et al. (2013), and Wilson et al. (2014)

### 2.2 HBV

HBV belongs to the Hepadnaviridae family of DNA viruses and efficiently encodes seven key proteins. Among these, the X protein (HBx), core protein (HBc), and HBe antigen (HBeAg) are most closely associated with the development of hepatocellular carcinoma (HCC) (Xu et al., 2017; Kong et al., 2020).

HBc interferes with the recognition of WD repeat sequence 46 (WDR46) by the E3 ubiquitin ligase TRIM25, thereby inhibiting its degradation via the ubiquitin-proteasome pathway. Inhibits its degradation via the ubiquitin-proteasome pathway. Elevated WDR46 further acts as a transcriptional coactivator, promoting c-Myc recruitment to the promoter region of nucleolar spindle-associated protein 1 (NUSAP1), thereby activating transcription of this proliferation-related gene. This significantly enhances hepatocellular carcinoma cell growth and migration both *in vitro* (HepG2 and Huh7) and at the animal level (subcutaneous transplanted tumor mice, lung metastasis mice) (Kong et al., 2025).

HBeAg and its precursor protein synergistically promote hepatocarcinogenesis through dual mechanisms: they disrupt the interaction between NUMB and p53, thereby blocking p53 nuclear translocation and activation, while simultaneously enhancing E3 ubiquitin ligase HDM2 -mediated degradation of p53 via the ubiquitin-proteasome pathway, thus systematically suppressing p53-dependent apoptosis (Liu et al., 2016); concurrently, HBeAg directly binds to IκB kinase regulatory subunit NEMO, inhibiting K63-linked polyubiquitin chain modification mediated by TNF receptor-associated factor 6 (TRAF6). This weakens IL-1β-induced NF-κB signaling activation, downregulates the host innate immune response, and creates favorable conditions for viral replication and persistent infection (Wang et al., 2019).

Furthermore, another key protein, HBx, plays a central role in tumor progression through distinct ubiquitin mechanisms. For instance, HBx recruits the cytoskeletal protein LASP1 and the E3 ubiquitin ligase SYVN1 to form a functional complex, promoting the ubiquitination and degradation of glutamate dehydrogenase 1 (GLUD1). This process releases GLUD1’s inhibitory effect on the AKT signaling pathway. Simultaneously upregulating the expression of the pro-oncogenic factor IL-32, thereby establishing a key pro-oncogenic axis linking metabolic reprogramming to inflammatory microenvironment remodeling (You et al., 2024); recent studies reveal that HBx recruits β-catenin to trans-activate the expression of the deubiquitinating enzyme USP26. USP26 stabilizes the NAD^+^-dependent deacetylase SIRT1 through deubiquitination, enhancing its post-transcriptional protein stability and oncogenic activity. This mechanism has been validated *in vitro* and in genetic and xenograft mouse models (Ma et al., 2024).

Notably, HBV can also reconfigure the regulatory logic of the host ubiquitin network, forming multiple self-reinforcing oncogenic cycles: for example, HBx abnormally enhances TGF-β signaling by blocking the ubiquitin-mediated degradation of the transcription factor SMAD4 in the TGF-β pathway, thereby positively feeding back to promote its own expression (Figure 2) (Chaomin et al., 2022); recent studies further reveal that HBx enhances transcription of DTL, a key component of the Cullin4 E3 ligase complex. This induces conformational changes that inhibit DTL’s own ubiquitin-mediated degradation, forming an HBx/DTL positive feedback loop that continuously drives hepatocellular carcinoma progression (Xie et al., 2025).

In HBV-associated hepatocellular carcinoma (HBV-HCC), manipulation of the SUMOylation network by viral proteins constitutes another crucial mechanism promoting viral replication and tumorigenesis. A recent observational study employed qPCR to detect SUMO gene expression in tumor and adjacent non-tumor tissues from 58 HBV-HCC patients. Results demonstrated significantly elevated SUMO2 expression in HCC tissues (*p* = 0.01), suggesting its potential involvement in malignant progression (Khai et al., 2024).

SUMO2 modification serves multiple functions throughout the HBV life cycle. HBc serves as a modification substrate for SUMO2, and its SUMOylation is a prerequisite for binding to the specific promyelocytic leukemia nuclear body (PML-NBS). By triggering viral capsid disassembly, HBc mediates rcDNA transport to the nucleus and influences cccDNA stability and transcriptional activity, leading to persistent HBV infection (Hofmann et al., 2023). Conversely, HBx promotes EMT and hepatocyte overgrowth by disrupting the SUMOylation status of E-cadherin (Ha et al., 2016). Studies indicate that HBx expression drives SUMO1 and SUMO2/3 binding to E-cadherin, leading to its degradation and loss of intercellular junctions. Notably, SUMOylation of E-cadherin exhibits subtype-specific regulation: SUMO2/3 activation is more pronounced in HepG2-HBx cells, whereas SUMO1 modification dominates in HBx transgenic mice (Ha et al., 2016).

HBx also reshapes the intracellular environment by regulating the SUMOylation status of host proteins. For example, HBx hijacks the Sp110 protein within PML nucleosomes, inducing its desumoylation and release from nucleosomes. The resulting Sp110–SENP1–HBx complex further relocates HBx to multiple target gene promoter regions, altering the host gene expression profile to promote viral replication and assist in evading immune surveillance (Sengupta et al., 2017). Furthermore, during chronic hepatitis B progression, HBx upregulates the expression of the centrosome-associated protein CPAP and enhances its SUMOylation through inflammatory signaling. This stabilizes the HBx protein in an NF-κB-dependent manner, forming a positive feedback loop that continuously activates NF-κB signaling, thereby driving hepatocyte transformation and hepatocellular carcinoma development (Yen et al., 2019).

In summary, multiple HBV-encoded proteins synergistically promote viral persistence and host cell malignant transformation by precisely regulating host ubiquitination and SUMOylation processes, further highlighting their pivotal role in HBV-associated hepatocellular carcinoma development (Table 2).

**Table 2:** Mechanism of HBV oncoprotein carcinogenesis via the ubiquitination and SUMOylation.

Viral protein	UPS/SUMO activity	Target	Main mechanism	References
HBV HBx	TRIM25 (E3 ligase)	WDR46	Promoting the recruitment of c-Myc to the promoter region of NUSAP1, activating the transcription of this gene, and enhancing the growth and migration ability of liver cancer cells	Kong et al. (2025)
HBV HBeAg	HDM2 (E3 ligase)	p53	Promotion of HDM2-mediated ubiquitination and degradation of p53 for apoptotic escape of hepatocytes	Liu et al. (2016)
	TRAF6 (E3 ligase)	NEMO (K63)	Down-regulate the host’s innate immune response to create favorable conditions for viral replication and persistent infection	Wang et al. (2019)
HBV HBx	SYVN1 (E3 ligase)	GLUD1	Degradation of the GLUD1, elevating IL-32 and enhancing tumor cell growth and migration	You et al. (2024)
	USP26 (deubiquitinase)	SIRT1	Enhancing USP26-SIRT1 binding, leading to stabilization of SIRT1 and driving hepatocarcinogenesis	Ma et al. (2024)
	Ubiquitination	SMAD4	Amplifying TGF-β signaling and upregulating HBx expression	Chaomin et al. (2022)
	Cullin4 (E3 ligase)	HBx	Inhibiting the ubiquitination degradation of HBx itself, forming a positive feedback loop to promote cancer	Xie et al. (2025)
	SUMOylation	E-cadherin	Promoting epithelial-mesenchymal transition (EMT) and hepatocyte overgrowth	Ha et al. (2016)
	SUMOylation	Sp110	Inducing its desumoylation and release from the nucleus to form the SP110-SENP1-HBX complex, locating HBx to the promoter region of the target gene, altering the host gene expression profile, promoting viral proliferation and assisting it in evaded immune surveillance	Sengupta et al. (2017)
	SUMOylation	CPAP	Enhancing NF-κB activation and promoting hepatocyte transformation	Yen et al. (2019)

### 2.3 HCV

HCV is an enveloped single-stranded positive-sense RNA virus and one of the primary causes of HCC (Yuan et al., 2025). HCV activates E3 ubiquitin ligase Itch, leading to polyubiquitination of AAA-type ATPase VPS4A, thereby enhancing its enzymatic activity and promoting HCV particle release (Deng et al., 2022). HCV upregulates E3 ubiquitin ligase PDLIM2, which specifically targets STAT2 for degradation via the ubiquitin-proteasome pathway, suppressing the host innate immune response and enabling HCV persistence in the host (Joyce et al., 2019). This persistent state and the chronic inflammation it induces constitute a major cause of HCC development (Yuan et al., 2025).

Beyond chronic inflammation, endoplasmic reticulum stress and DNA damage also contribute to HCV-associated HCC progression (Yuan et al., 2025). Ubiquitin-conjugating enzyme E2S (UBE2S) interacts with HCV-encoded RNA-dependent RNA polymerase NS5A and mediates NS5A degradation via a K11-linked polyubiquitin chain-mediated proteasome-dependent pathway. However, in HCV-infected cells, UBE2S is downregulated by HCV-induced endoplasmic reticulum stress, allowing NS5A to remain stable and ensure smooth viral replication (Pham et al., 2019). Furthermore, NS5A stabilizes RAD51AP1 by modulating the ubiquitin-proteasome pathway, disrupting the formation of the RAD51/RAD51AP1/UAF1 trimeric complex and impairing DNA repair in HCV-infected cells (Figure 2) (Nguyen et al., 2018). Thus, by manipulating the ubiquitin-proteasome pathway, HCV achieves persistent infection while directly inducing genomic instability in host cells, thereby establishing critical conditions for hepatocyte malignant transformation.

Furthermore, HCV utilizes ubiquitin modification to mark its own proteins, promoting viral replication and even determining viral host tropism. HCV employs K63-linked polyubiquitin chains to modify its NS2 protein, which has been identified as a signal for HCV assembly (Barouch-Bentov et al., 2016). The HCV-encoded RNA-dependent RNA polymerase NS5B directly interacts with the E3 ubiquitin ligase TRIM26, mediating K27-linked polyubiquitin chain modification at its K51 site. This non-degradative ubiquitination enhances the interaction between NS5B and another critical viral protein, NS5A, thereby effectively promoting HCV genome replication (Liang et al., 2021). The study further revealed that mouse-derived TRIM26, due to a unique six-amino-acid insertion in its protein sequence, fails to bind effectively to NS5B and thus cannot support HCV replication. Conversely, ectopic expression of human TRIM26 in mouse hepatocellular carcinoma cells—which inherently lack HCV infectivity—sufficiently restores an efficient viral replication cycle. This discovery not only reveals TRIM26 as a key host determinant for HCV’s interspecies infection but also highlights HCV’s sophisticated strategy of hijacking host-specific ubiquitin modification mechanisms to precisely regulate viral protein functions, thereby achieving efficient self-replication and proliferation (Liang et al., 2021).

The HCV core protein is one of the virus’s most important structural proteins and also a multifunctional protein involved in viral pathogenesis and hepatocellular carcinoma development (Vescovo et al., 2016). The HCV core protein inhibits the expression of DNA methyltransferase and E6-associated protein through DNA methylation, thereby protecting itself from degradation by the ubiquitin-proteasome pathway and stimulating viral proliferation (Kwak et al., 2016). Research indicates that HCV core protein also downregulates p16 levels via ubiquitin-proteasome pathway degradation, releasing its inhibition on CDK4/6 and stimulating human hepatocellular carcinoma cell growth (Figure 2) (Cha et al., 2021). Another study demonstrates that HCV core protein disrupts monoubiquitination of histone H2A K119 at Homeobox (HOX) gene promoters, inducing abnormal HOX gene expression and participating in carcinogenesis (Kasai et al., 2021).

Research has revealed that the HCV core protein can also promote malignant tumor progression by interfering with the host SUMOylation pathway. For instance, it mediates the SUMOylation modification of the metastasis-inhibiting protein Nm23-H1 and promote its degradation, thereby promoting its degradation and enhancing the migration and invasion capabilities of hepatocellular carcinoma cells (Figure 2) (Paul et al., 2019). This suggests that the SUMOylation pathway may represent one potential link in the oncogenic network constructed by the HCV core protein.

**Figure 3:**
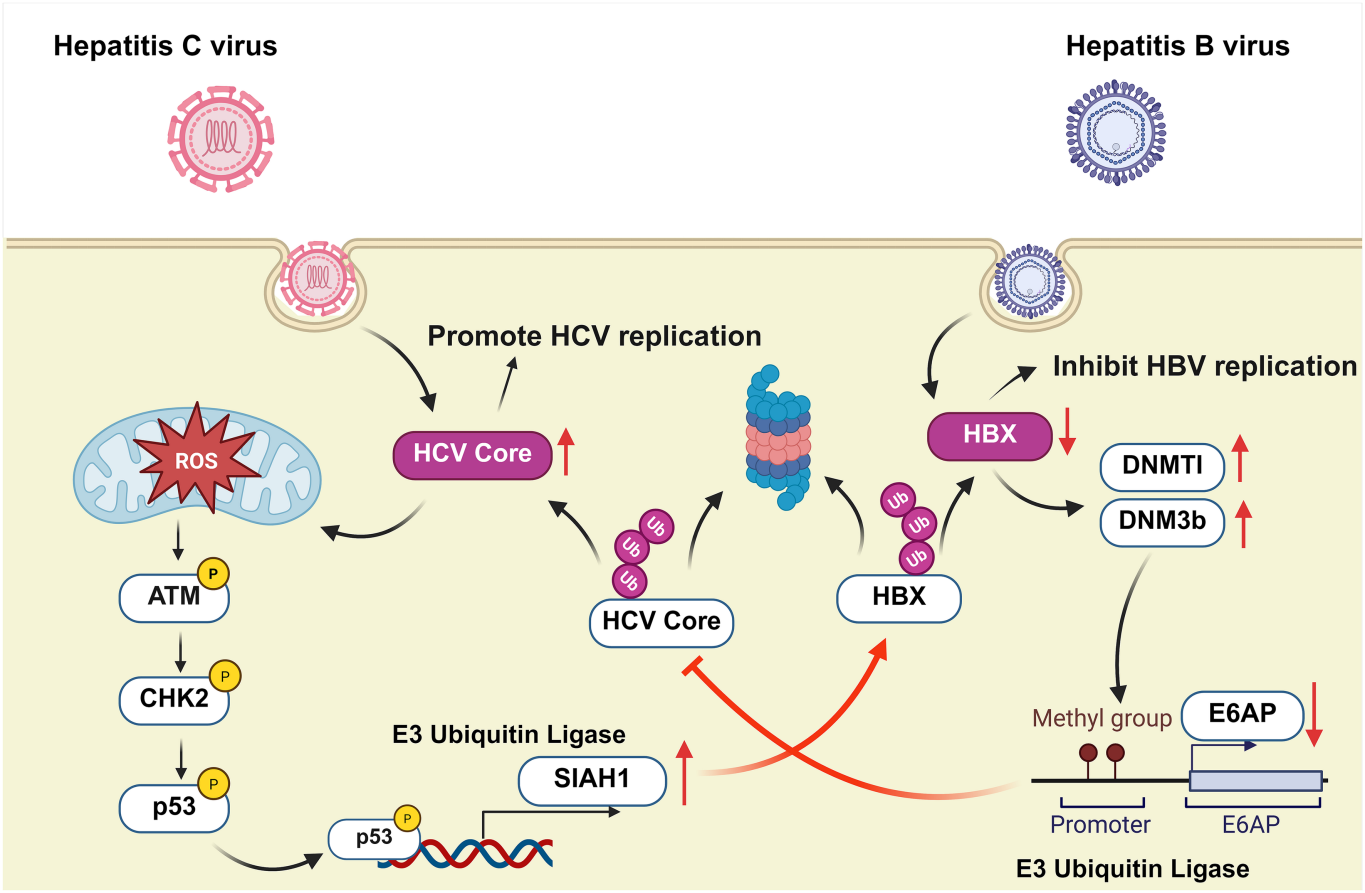
Bidirectional regulatory mechanisms in HBV and HCV co-infection. Left: HCV core protein induces mitochondrial reactive oxygen species (ROS) production, activating the ATM-Chk2 DNA damage response pathway and promoting p53 upregulation. p53 then transcribes and activates the E3 ubiquitin ligase SIAH1, which mediates the ubiquitination and degradation of HBx—a key HBV replication regulator—ultimately inhibiting HBV replication. Right: HBx elevates the protein levels and activity of DNMT1/DNMT3b, catalysing DNA methylation within the E6AP promoter region. This inhibits E6AP transcription, thereby impeding the ubiquitination and degradation of the HCV core protein. Consequently, the enhanced stability of the core protein promotes increased HCV replication efficiency (Lee et al., 2021; Yoon et al., 2022).

Notably, due to similar transmission routes, HBV and HCV co-infection is common (Liu and Chen, 2022). Although both HBx and HCV core protein exhibit potent oncogenic capabilities in independent infections, their interactions during co-infection reveal intriguing asymmetry (Figure 3). HBx protects HCV core protein from degradation via the E6AP-mediated ubiquitin-proteasome pathway, while HCV core protein downregulates HBx levels through the ubiquitin-proteasome pathway mediated by the E3 ubiquitin ligase Siah-1 and the ubiquitin ligase complex (Table 3) (Yoon et al., 2022; Lee et al., 2021).

**Table 3:** Mechanism of HCV oncoprotein carcinogenesis via the ubiquitination and SUMOylation.

Viral protein	UPS/SUMO activity	Target	Main mechanism	References
HCV	Itch (E3 ligase)	VPS4A	Leading to the polyubiquitination of VPS4A, enhancing its enzymatic activity, and promoting the release of HCV particles	Deng et al. (2022)
	PDLIM2 (E3 ligase)	STAT2	Inhibiting the host’s innate immune response, leading to the persistent presence of HCV in the host	Joyce et al. (2019)
HCV NS5A	UBE2S (E2 ligase)	NS5A (K11)	UBE2S is downregulated by endoplasmic reticulum stress caused by HCV, allowing NS5A to exist stably and ensuring the smooth progress of viral replication	Pham et al. (2019)
	Ubiquitination	RAD51AP1	Stabilization of RAD51AP1 via the UPS interferes with DNA repair complex formation and disrupts DNA repair	Nguyen et al. (2018)
HCV NS2	Ubiquitination	NS2 (K63)	Ubiquitination is used to label one’s own protein to promote viral proliferation and even determine the host orientation of the virus	Barouch-Bentov et al. (2016)
HCV NS5B	TRIM26 (E3 ligase)	NS5B (K27)	Enhancing the interaction between NS5B and the virus NS5A, thereby effectively promoting the replication of the HCV genome	Liang et al. (2021)
HCV Core protein	E6AP (E3 ligase)	E6AP	Inhibiting the expression of E6AP, protect itself from proteasome degradation, and stimulating the reproduction of the virus	Kwak et al. (2016)
	Ubiquitination	p16	Alleviating its inhibitory effect on CDK4/6 and promoting the proliferation of human hepatocellular carcinoma cells	Cha et al. (2021)
	Ubiquitination	Histone H2A (K119)	Resulting in aberrant HOX gene expression and contributing to malignant transformation	Kasai et al. (2021)
	SUMOylation	Nm23-H1	Promoting SUMO-chemical degradation and transcriptional down-regulation of the Nm23-H1 enhancing hepatocellular carcinoma cell migration and invasion	Paul et al. (2019)

### 2.4 EBV

During the establishment of latent infection in B cells, EBV exhibits four distinct latent forms: latent phase III, latent phase II, latent phase I, and latent phase 0, each contributing uniquely to carcinogenesis. Latent phase III expresses all six latent genes and is associated with diffuse large B-cell lymphoma in severely immunocompromised individuals. Latent phase II primarily expresses EBNA1 and LMPs, correlating with malignancies such as nasopharyngeal carcinoma, where LMP1 plays a pivotal role in regulating cell growth and apoptosis. Latent phase I expresses only EBNA1, a protein that helps maintain EBV genomic stability and is closely associated with the development of cancers like Burkitt’s lymphoma (Xiao et al., 2025).

EBV enhances its latent persistence and oncogenic potential in host cells by regulating the host ubiquitin-SUMO network through latent proteins like LMP1 and EBNA3C. Specifically, LMP1 activates the NF-κB pathway, promoting MDM2-p53 interaction that leads to p53 ubiquitination and degradation. Concurrently, LMP1 enhances Bcl-2 expression, thereby promoting proliferation and inhibiting apoptosis in lymphoma cells (KHYG-1) (Zeng et al., 2020).

Interferon regulatory factor 7 (IRF7) is a key regulator in the host’s innate antiviral immune response to type I interferons (IFNs). LMP1 activates IRF7 through TRAF6-/RIP-dependent K63 polyubiquitination (Ning et al., 2011). Concurrently, LMP1 promotes TRAF6 polyubiquitination or induces TRAF1 ubiquitination, thereby recruiting the LUBAC complex. This complex plays a central role in LMP1-mediated NF-κB activation and IRF7 regulation: while LUBAC recruitment enhances NF-κB pathway activation, it simultaneously negatively regulates LMP1-enhanced IRF7 transcriptional activity. Notably, the LMP1-interacting subunit RNF31 within LUBAC exhibits significantly higher expression levels in latent phase III cells compared to latent phase I (Wang et al., 2017). Furthermore, the LMP1-induced deubiquitinating enzyme A20 negatively regulates this process by removing ubiquitin modifications from IRF7, thereby impacting innate immune responses (Figure 2) (Ning and Pagano, 2010). These mechanisms collectively form a bidirectional “activation-inhibition” equilibrium system. Disruption of IRF7 signaling homeostasis triggers chronic inflammation and abnormal type I interferon responses, ultimately driving tumorigenesis and progression by inducing genomic instability or promoting immune escape.

Studies indicate that high levels of SUMOylation in host proteins are often closely associated with tumorigenesis (Seeler and Dejean, 2017). In Epstein–Barr virus (EBV), LMP1 regulates SUMOylation through two primary mechanisms: First, its CTAR3 domain directly binds to SUMO ligase Ubc9, thereby promoting SUMOylation of intracellular proteins and enhancing tumor cell migration; Second, it activates the NF-κB pathway through its CTAR1/CTAR2 domains, thereby upregulating SUMO and related regulatory proteins (Bentz et al., 2011; Salahuddin et al., 2019). These two mechanisms synergistically lead to abnormal SUMOylation levels in key signaling pathways, ultimately driving tumor-associated phenotypes. Additionally, the study identified four EBV proteins that upregulate SUMOylation levels (SM, BGLF2, BMRF1, BVRF2) and one that downregulates SUMOylation (BRLF1) (De La Cruz-Herrera et al., 2018). These findings not only expand our understanding of EBV-encoded protein functions but also reveal novel mechanisms by which the virus influences tumorigenesis through regulation of the host SUMOylation system.

EBNA3C, another crucial latent-phase protein of EBV, plays a key role in ubiquitin-dependent regulation. Experiments conducted in BJAB and SAOS-2 cell lines revealed that EBNA3C targets pRB through the E3 ubiquitin ligase SCF^Skp2^, promoting its ubiquitin-proteasome-dependent degradation. Notably, the functional region of EBNA3C mediating pRB degradation exhibits sequence similarity to the HPV E7 protein (Knight et al., 2005). Furthermore, EBNA3C similarly utilizes SCFSkp2 to induce degradation of another tumor suppressor protein, RASSF1A, thereby upregulating Cyclin D1/E expression. It also relieves proteasomal degradation of the oncogene Pim-1, subsequently inhibiting p21 activity and ultimately enhancing the proliferative capacity of EBV-transformed cells (Banerjee et al., 2014; Zhang et al., 2019). Regarding SUMOylation, studies reveal that in EBV-transformed lymphoblastoid cell lines, EBNA3C interacts with SUMO-1 and SUMO-3, thereby synergistically activating the LMP1 promoter with EBNA2 to enhance its expression (Rosendorff et al., 2004).

The carcinogenic effects of EBV strictly depend on the specific expression patterns of viral proteins during different latent phases: for instance, EBNA3C is predominantly enriched in latent phase III, whereas LMP1 is expressed in both latent phases II and III (Münz, 2025). Based on EBV’s stage-specific expression patterns, we hypothesise that during latent-to-active phase transitions, oncogenic effects mediated by ubiquitin/SUMOylation of viral proteins in the preceding stage cease with the termination of that protein’s expression. However, the new phase initiates novel viral protein-dominated ubiquitin-mediated regulation (such as modification of other oncogenic targets). This dynamic pattern of “replacing old effects with new ones” enables EBV to continuously drive malignant cellular phenotypes through the ubiquitin system, rather than interrupting the carcinogenic process, ultimately promoting tumorigenesis (Table 4).

**Table 4:** Mechanism of EBV oncoprotein carcinogenesis via the ubiquitination and SUMOylation.

Viral protein	UPS/SUMO activity	Target	Main mechanism	References
EBV LMP1	MDM2 (E3 ligase)	p53	Promoting the degradation of p53, enhancing the expression of Bcl-2, thereby promoting the growth of lymphoma cells and inhibiting cell apoptosis	Zeng et al. (2020)
	TRAF6 (E3 ligase)	IRF7 (K63)	Activating IRF7 and inducing type I interferon production	Ning et al. (2011)
	TRAF6, TRAF1	LUBAC	Enhancing NF-κB pathway activity, inhibiting IRF7 and inducing type I interferon production	Wang et al. (2017)
	A20 (deubiquitinase)	IRF7	Activating IRF7 and inducing type I interferon production	Ning and Pagano (2010)
	Ubc9 (E2 ligase)	Intracellular proteins	The CTAR3 domain binds to Ubc9, promoting SUMOylation modification of intracellular proteins and enhancing tumor migration ability. The CTAR1/CTAR2 domain activates the NF-κB pathway, upregulates the expression of SUMO and related regulatory proteins, and drives the formation of tumor-related phenotypes	Bentz et al. (2011) and Salahuddin et al. (2019)
EBV EBNA3C	SCF^Skp2^ (E2 ligase)	pRB, RASSF1A, Pim-1	Inducing degradation of the oncogenic protein RASSF1A and rescuing degradation of the oncogenic protein Pim-1, promoting the proliferation ability of EBV-transformed cells	Knight et al. (2005), Banerjee et al. (2014), and Zhang et al. (2019)
	SUMO-1, SUMO-3	LMP1 promoter	Promoting the expression of LMP1 and enhancing its carcinogenic activity	Rosendorff et al. (2004)

### 2.5 Kaposi’s sarcoma-associated herpesvirus

#### 2.5.1 Latent infection

The latent infection state of Kaposi’s sarcoma-associated herpesvirus (KSHV) is primarily regulated by the latent-associated nuclear antigen (LANA). In ubiquitin-mediated regulation, LANA assembles specific E3 ubiquitin ligase complexes to promote the proteasomal degradation of multiple tumor suppressor proteins (such as p53, VHL, and NF-κB) (Cerqueira et al., 2016; Cai et al., 2006). Additionally, LANA upregulates Aurora A kinase, enhancing the interaction between p53 and LANA, thereby accelerating the ubiquitin-proteasome degradation pathway of p53 (Cai et al., 2012). Notably, LANA also possesses the function of sequestering E3 ubiquitin ligases to protect certain oncoproteins. For example, LANA recruits E3 ubiquitin ligases such as RLIM and FBW7 away from their physiological targets, thereby interfering with the ubiquitin-proteasome degradation pathway of oncoproteins like TRF1, MCL-1, and ICN, ultimately promoting tumorigenesis and progression (Sun et al., 2014; Tadmor et al., 2020; Kim et al., 2021). Regarding SUMOylation regulation, LANA suppresses the expression of the deSUMOylase SENP6, thereby reducing the production of SUMOylated LANA and exerting negative feedback regulation on its downstream oncogenic pathways (Lin et al., 2017).

#### 2.5.2 Lytic phase

K-RTA is a switch protein that regulates the transition of KSHV from latent to lytic replication. It possesses SUMO-targeting ubiquitin ligase (STUbL) activity, enabling it to specifically recognise SUMO-modified proteins via its own SUMO interaction motif (SIM). Utilising the ubiquitin ligase function of its RING-like domain, it primarily mediates polyubiquitination with K48- and K63-linked chains, ultimately directing the proteasomal degradation of its substrates (Causey et al., 2025). Functionally, K-RTA targets the SUMO-modified host protein PML, promoting its degradation via the ubiquitin-proteasome pathway, thereby aiding viral escape from the host’s innate immune response (Campbell and Izumiya, 2012). Moreover, it can directly or indirectly ubiquitinate and degrade multiple host or viral inhibitory factors, thereby clearing obstacles to viral lysis and reactivation. For instance, K-RTA suppresses innate immune signaling pathways by ubiquitinating myeloid differentiation factor 88 (Myd88) and enhancing its proteasome-dependent degradation, thus creating favorable conditions for viral replication (Zhao et al., 2015).

**Figure 4:**
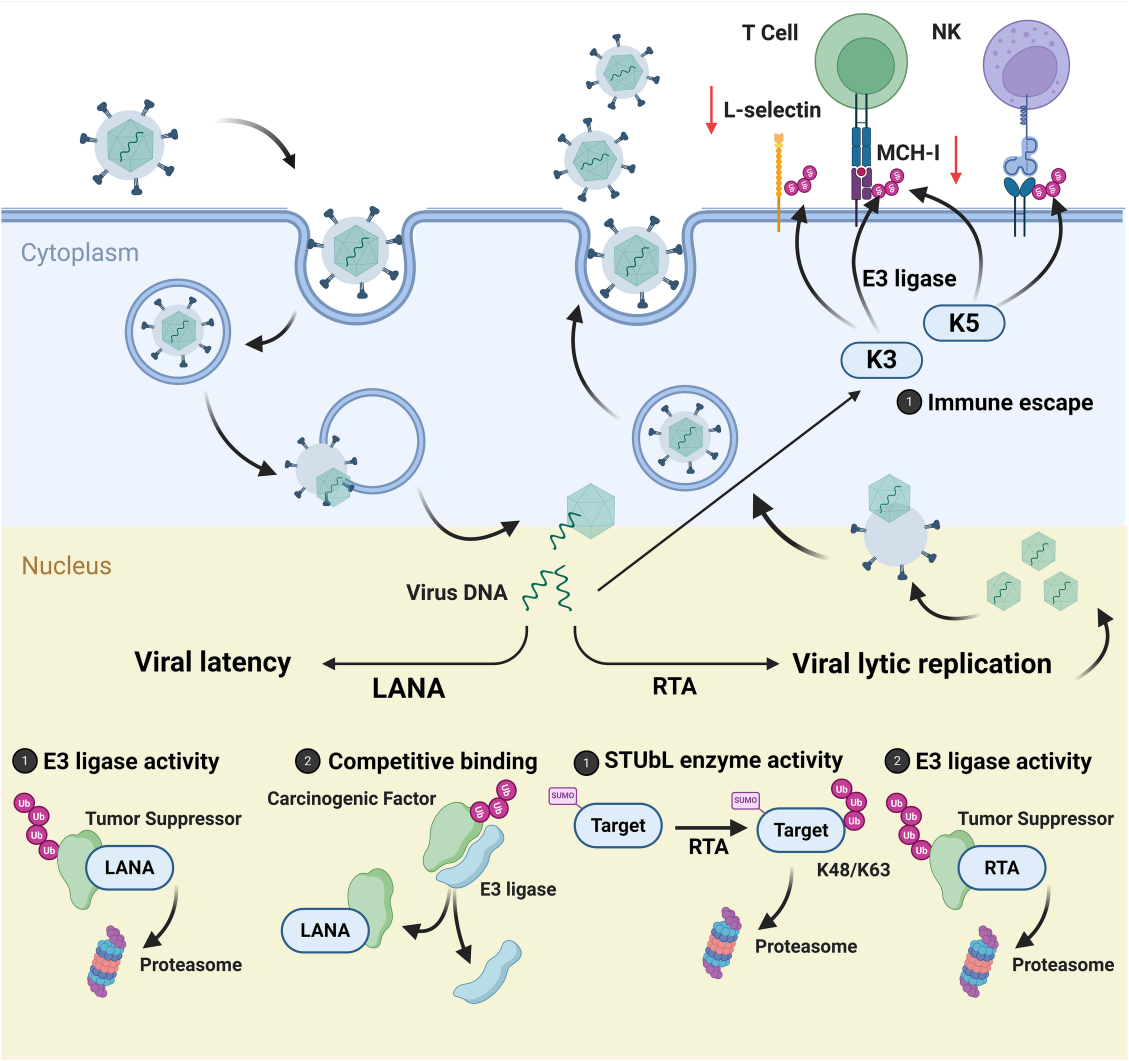
Mechanisms of KSHV-associated protein ubiquitination/SUMOylation in tumor regulation. This diagram illustrates the core biological processes following herpesvirus infection of host cells: ① Viral particles enter the host cytoplasm via endocytosis, initiating infection; the viral life cycle comprises a latent phase (maintained by LANA to preserve the viral DNA in a nuclear circular latent state) and a lytic replication phase (driven by RTA) (Taverna and Franchi, 2019); ② Viral proteins regulate host and self-proteins through ubiquitin modification: LANA mediates the proteasomal degradation of tumor suppressor factors via its E3 ligase activity, while competitively binding oncogenes (Cerqueira et al., 2016; Cai et al., 2006; Sun et al., 2014; Tadmor et al., 2020; Kim et al., 2021); RTA possesses STUBL enzyme activity (Causey et al., 2025); RTA also degrades tumor suppressor factors via E3-mediated enzyme activity (Zhao et al., 2015); The virus-encoded K3/K5 (E3 ligase) downregulates MHC-I and E-selectin molecules on the host cell membrane surface, thereby impeding recognition and killing by T cells and NK cells to achieve immune evasion (Brulois et al., 2014; Kajikawa et al., 2021; Nanes et al., 2017).

KSHV encodes two E3 ubiquitin ligases, K3 and K5, expressed during the viral lytic replication phase. These enzymes synergistically promote immune evasion and tumorigenesis by regulating the stability of multiple host proteins. Both K3 and K5 induce ubiquitin-proteasome pathway degradation of MHC-I molecules, aiding infected cells in evading recognition and clearance by CD8^+^ T cells (Figure 2) (Brulois et al., 2014). K5·dependent·RING-domain·E2·centred on the tryptophan residue at the binding site, modifies the lysine residues in the cytoplasmic domain of L-selectin with polyubiquitin chains. This facilitates ubiquitin-dependent endocytosis, promoting L-selectin degradation and thereby downregulating its expression on the cell surface. Consequently, this further enhances viral escape from the host’s innate immune response (Kajikawa et al., 2021). K3 circumvents the antiviral immune response mediated by the lymphotoxin receptor (LTR) by interfering with the glycosylation process and inhibiting the membrane transport of lymphotoxin β receptor ligand (LTB) (Kang et al., 2025). Beyond immune regulation, K5 also ubiquitinates E-cadherin, driving its endocytosis and degradation, thereby disrupting endothelial cell junctions and barrier integrity. This mechanism is considered to play a significant role in tumor invasion and metastasis (Nanes et al., 2017). In summary, KSHV ingeniously hijacks the host ubiquitinylation network by encoding its own E3 ubiquitin ligase, transforming the cell’s intrinsic homeostasis regulatory mechanisms into tools for achieving immune evasion and malignant transformation. This highly sophisticated strategy of molecular parasitism not only reveals a novel paradigm for viral carcinogenesis but also provides a potential pathway for developing precision therapies targeting the virus’s own E3 ubiquitin ligase (Figure 4 and Table 5).

**Table 5:** Mechanism of KSHV oncoprotein carcinogenesis via the ubiquitination and SUMOylation.

Viral protein	UPS/SUMO activity	Target	Main mechanism	References
KSHV LANA	Ubiquitination	p53, VHL, NF-κB	Assembly of specific E3 ubiquitin ligase complexes to degrade multiple oncogenic proteins	Cerqueira et al. (2016) and Cai et al. (2006)
	RLIM, FBW7 (E3 ligase)	TRF1, MCL-1, ICN	Isolation of the E3 ligases RLIM and FBW7 prevents normal ubiquitination degradation of their target proteins, thereby promoting the occurrence and progression of tumors	Sun et al. (2014), Tadmor et al. (2020), and Kim et al. (2021)
	SENP6 (SUMO-specific proteases)	SENP6	Inhibition of the expression of the SENP6 reducing the formation of SUMOxylated LANA and producing negative feedback regulation of downstream pathways	Lin et al. (2017)
KSHV K-RTA	K-RTA (E3 ligase)	PML, Myd88	Suppressing innate immune signaling pathways creates favorable conditions for viral replication	Campbell and Izumiya (2012) and Zhao et al. (2015)
KSHV K3	K3 (E3 ligase)	MHC-I, LTB	Inducing degradation of MHC-I molecules to escape T-cell recognition; inhibiting membrane transport of LTB by interfering with glycosylation and circumvents LTR-mediated antiviral immunity	Brulois et al. (2014) and Kang et al. (2025)
KSHV K5	K5 (E3 ligase)	MHC-I, NK cell ligands, E-cadherin	Down-regulation of MHC-I, NK cell ligand and I-selectin by ubiquitination endocytosis to aid immune escape; ubiquitination of E-calmodulin to disrupt cell junctions, promoting tumor invasion and metastasis	Brulois et al. (2014) and Nanes et al. (2017)

### 2.6 Human T-cell leukemia virus type 1

Human T-cell leukemia virus type 1 (HTLV-1) is the key pathogen causing adult T-cell leukemia/lymphoma (ATL). Its encoded Tax protein, as a crucial viral oncoprotein, hijacks the host ubiquitin and SUMO modification systems to continuously activate the NF-κB signaling pathway, thereby driving tumorigenesis (Lavorgna and Harhaj, 2014). The Tax protein possesses intrinsic E3 ubiquitin ligase activity, capable of catalysing the formation of diverse mixed ubiquitin chain modifications including K6, K11, K27, K29, K33, K48, and K63. It binds to NEMO, thereby directly activating IκB kinase (Wang et al., 2016). This process further maintains the stability of the Tax protein itself through a positive feedback mechanism, forming a continuously activated signaling loop (Millen et al., 2020). Within NF-κB-dependent carcinogenic mechanisms, Tax further inhibits the phosphorylation of YAP by LATS1, a kinase within the Hippo signaling pathway, while simultaneously blocking YAP degradation via the ubiquitin-proteasome pathway. This results in the accumulation of YAP within the nucleus, where it drives malignant proliferation of T cells and promotes cancer development (Zhao T. et al., 2022). Moreover, the oncogenic activity of Tax is finely regulated by the ubiquitin-like modifier Urm1. Studies in HTLV-I-transformed HuT-102 cells revealed that Urm1 covalently binds to Tax via its lysine residues (sites 4–8), facilitating Tax translocation to the cytoplasm. This enhances Tax’s capacity to activate NF-κB, thereby promoting cancer progression (Hleihel et al., 2018). In summary, Tax undergoes both ubiquitination and SUMOylation, which not only sustain NF-κB signaling and maintain its own stability but also amplify downstream oncogenic signals, ultimately leading to uncontrolled cell proliferation and virus-mediated tumorigenesis (Table 6).

**Table 6:** List of other carcinogenic microorganisms by which speculative oncogenic microorganisms manipulate ubiquitination and SuMOylation pathways to drive tumors.

Carcinogenic microorganisms	Protein	UPS/SUMO Activity	Target	Main mechanism	References
HTLV-1	Tax	Tax (E3 ligase)	IKK	Activation of NF-kB signaling and evasion of the immune response	Wang et al. (2016)
		Ubiquitination	LATS1	Blocking the ubiquitination and degradation of YAP, promoting the nuclear accumulation of YAP and driving the malignant proliferation of T cells	Zhao T. et al. (2022)
MCPyV	LT	Ubiquitination	LT	Limiting viral replication and cell growth, facilitating viral latency	Nwogu et al. (2020a)
	sT	Fbw7 (E3 ligase)	LT	Stabilizing LT, c-Myc and Cyclin E, promoting cellular proliferation	Kwun et al. (2013)
		Fbw7 (E3 ligase)	MMP-9 Snail	Activation of MMP-9 and Snail, enhancing cellular migration and invasion	Nwogu et al. (2020b)
*Helicobacter pylori* (*Hp*)	CagA	TRAF6 (E3 ligase) Ubc13 (E2 ligase)	TAK1 (K63)	Enhancing the kinase activity of TAK1, activating the NF-κB pathway, trigger chronic inflammation and promoting the occurrence of gastric cancer	Lamb et al. (2013) and Lamb et al. (2009)
		TRIP12 (E3 ligase)	p14ARF, p53 (K48)	Accelerating the degradation of p53 proteasome and weakening the tumor suppressive function of p53	Horvat et al. (2018) and Wei et al. (2010)
		XIAP (E3 ligase)	Siva1	Promoting the ubiquitination and degradation of the apoptotic factor Siva1 and reducing the apoptosis of DNA-damaged cells	Palrasu et al. (2020)
		Ubiquitination	PD-L1	Enhancing the stability of PD-L1, inhibits T cell activity, and promoting immune escape in gastric cancer	Liu et al. (2025)

### 2.7 Merkel cell polyomavirus

Merkel cell polyomavirus (MCPyV) is the only known polyomavirus capable of directly causing human cancer (Loke et al., 2022). Its oncogenic potential is associated with the virus-encoded large T antigen (LT) and small T antigen (sT), which synergistically interfere with the host ubiquitin-proteasome system to regulate viral replication and drive cellular malignant transformation. LT is a key protein in viral replication, whose MUR domain contains multiple sites interacting with E3 ubiquitin ligases. This domain mediates LT’s degradation via the ubiquitin-proteasome pathway, thereby limiting viral replication and cellular growth—a mechanism potentially advantageous for establishing latent infection (Nwogu et al., 2020a). In contrast, sT stabilizes LT via its LT stabilizing domain (LSD), inhibiting the activity of the E3 ubiquitin ligase SCF(Fbw7) and preventing degradation of its substrate LT. This stabilizes LT and cyclin proteins such as c-Myc and Cyclin E, promoting abnormal cellular proliferation (Kwun et al., 2013). The disruption of the host ubiquitination pathway by sT extends beyond this. By targeting FbW7 through LSD, it activates matrix metalloproteinase-9 (MMP-9) and EMT-related factors such as Snail, significantly enhancing cell migration and invasion capabilities. This may represent one of the key molecular foundations for the highly invasive nature of Merkel cell carcinoma (MCC) (Table 6) (Nwogu et al., 2020b).

### 2.8 Helicobacter pylori

Chronic *Hp* infection is a major risk factor for peptic ulcer disease and gastric cancer (Sokolova and Naumann, 2021). Among *Hp*’s multiple virulence factors, CagA modulates the host ubiquitin network to activate multiple signaling pathways that promote tumorigenesis, with NF-κB pathway activation being particularly critical (Taniguchi and Karin, 2018). Upon CagA injection into host cells via the type IV secretion system (T4SS), it directly binds to transforming growth factor-β-activated kinase 1 (TAK1) within host cells. CagA further recruits the RING-type E3 ubiquitin ligase TRAF6 and, through the synergistic action of the E2 ubiquitin ligase Ubc13 and its cofactor Uev1A, catalyzes the formation of K63-linked polyubiquitin chains on TAK1. This modification enhances TAK1’s kinase activity, thereby activating the canonical NF-κB signaling pathway, ultimately triggering persistent inflammatory responses and driving the progression of gastric cancer (Lamb et al., 2013; Lamb et al., 2009).

In addition to the aforementioned mechanisms, CagA also promotes the ubiquitin-proteasome pathway degradation of the tumor suppressor protein p14ARF, a process mediated by the HECT-type E3 ubiquitin ligase TRIP12 (Horvat et al., 2018). The degradation of p14ARF releases its inhibitory effect on HDM2, thereby promoting HDM2 phosphorylation at Ser166 and its subsequent activation. Activated HDM2 then enhances K48-linked ubiquitin chain modification of p53, leading to its degradation via the proteasome pathway and significantly impairing its tumor-suppressing function (Wei et al., 2010). Similarly, studies on *Helicobacter pylori* strain B128 and its carcinogenic derivative strain 7.13 revealed that CagA further enhances the E3 ubiquitin ligase activity of XIAP—a member of the IAP family—by activating its phosphorylation. This subsequently promotes the ubiquitin-proteasome pathway degradation of the pro-apoptotic factor Siva1. This process suppresses apoptosis in DNA-damaged cells, thereby creating conditions conducive to malignant cellular transformation (Palrasu et al., 2020). Recent studies also reveal CagA’s role in immune evasion. Experiments in AGS and MFC cell lines demonstrate that CagA upregulates squalene epoxidase (SQLE) expression, thereby significantly reducing PD-L1 ubiquitination levels and enhancing its protein stability. *In vivo* experiments further validated this pathway’s significance: when equal amounts of MFC cells were injected into BALB/c nude mice and C57BL/6 mice, tumor volumes were significantly larger in nude mice due to immune system differences. However, under conditions of CagA and SQLE overexpression, the tumor volume disparity between the two mouse strains disappeared, demonstrating the critical role of the CagA–SQLE–PD-L1 axis in promoting gastric cancer progression and immune evasion (Table 6) (Liu S. et al., 2025).

## 3 Mechanisms by which putative carcinogenic microorganisms manipulate ubiquitinylation and SUMOylation pathways to drive tumorigenesis

Within the intricate genealogy of microbial-cancer interactions, there remains a substantial category of pathogens whose associations remain speculative (Picardo et al., 2019; Isaguliants et al., 2021; Lauricella et al., 2025). Currently, research on HIV and the ubiquitin-SUMO modification systems has largely centred on their roles in viral latency, replication, and immune regulation (Zhang et al., 2021; Raja et al., 2022; Imbert and Langford, 2021; Zhou et al., 2020; Li et al., 2023). For instance, HIV can hijack the host’s ubiquitination and SUMOylation modification systems to facilitate immune evasion. Research indicates that the HIV-encoded Vpr protein recruits the E3 ubiquitin ligase complex DCAF1-CUL4A, mediating the ubiquitination and degradation of the transcription factor PU.1. This suppresses the immune response function of macrophages, thereby creating a favorable microenvironment for tumorigenesis (Virgilio et al., 2024). Furthermore, HIV infection can globally reduce the SUMOylation levels of host proteins and, by downregulating the expression of the UBA2 subunit of the SUMO E1 activator, further impairs immune surveillance functions and increases carcinogenic risk (Mete et al., 2022). The HIV Nef protein recruits E6AP to mediate the ubiquitin-proteasome pathway degradation of p53, thereby blocking apoptosis and promoting the survival of infected cells (Ali et al., 2020). HIV’s extensive disruption of these regulatory systems constitutes a persistent, systemic disruption of host cellular homeostasis, which is likely a key mechanism underpinning the high incidence of cancer in HIV-infected individuals (Table 7).

**Table 7:** List of mechanisms by which speculative oncogenic microorganisms manipulate ubiquitination and SUMOylation pathways to drive tumors.

Speculative oncogenic microorganisms	Viral protein	UPS/SUMO activity	Target	Main mechanism	References
HIV	Vpr	DCAF1-CUL4A (E3 ligase)	PU.1	Suppressing macrophage immune responses and creating a microenvironment favorable for tumorigenesis	Virgilio et al. (2024)
		UBA2 (E1 ligase)		Reducing the SUMOylation of host proteins weakening the immune surveillance function and increasing the risk of cancer	Mete et al. (2022)
	Nef	E6AP	p53	Degradation of the tumor suppressor p53, Blocking apoptosis and maintaining the survival of infected cells	Ali et al. (2020)
RSV	G	DZIP3 (E3 ligase)	GBP5	Degradation of the GBP5 to evade immune surveillance	Li et al. (2020)
SARS-CoV-2	nsp3	CBL (E3 ligase)	NF-κB	Suppressing NF-κB activation and modulating host inflammatory responses	Xie et al. (2024)
*Legionella pneumophila*	LotA	LotA (deubiquitinase)		Specific cleavage of the K6 ubiquitin chain interferes with immune signals	Schubert et al. (2020)
*E. coli*	NleG6	Ubiquitination	BRISC	Promoting the autophagic degradation of the BRISC complex to inhibit the NF-κB inflammatory response and regulate tumor progression	Pan et al. (2025)
Intestinal bacteria	Agantine	Rnf128 (E3 ligase)	β -catenin	Inhibiting the ubiquitination of β -catenin, activating the Wnt signaling pathway, and promoting the carcinogenesis of colon cancer	Lu et al. (2024)

Furthermore, the G protein of respiratory syncytial virus (RSV) induces upregulation of the host E3 ubiquitin ligase DZIP3, thereby promoting the ubiquitination and degradation of the interferon effector protein GBP5, thus evading host immune surveillance (Table 7) (Li et al., 2020). Similarly, the SARS-CoV-2 nsp3 protein recruits the host E3 ubiquitin ligase CBL via its characteristic SUD domain, thereby promoting the polyubiquitination of NEMO and subsequent proteasomal degradation. This inhibits the activation of the NF-κB pathway, thereby regulating the host inflammatory response (Table 7) (Xie et al., 2024). Although these viruses have not yet been definitively identified as direct carcinogens, their precise manipulation of the ubiquitin system demonstrates their capacity to indirectly influence host cell homeostasis and potentially contribute to carcinogenesis by disrupting key immune surveillance mechanisms and inflammatory balance. These findings suggest that they may be considered a novel class of indirect oncogenic agents in future, with their long-term carcinogenic risks warranting further attention and investigation.

Moreover, bacteria can exert synergistic oncogenic effects through the ubiquitin network. For instance, the LotA protein secreted by *Legionella pneumophila* belongs to the ovarian tumor (OTU) superfamily of deubiquitinating enzymes, capable of specifically cleaving K6-linked ubiquitin chains, thereby disrupting host immune signaling pathways (Schubert et al., 2020). The *E. coli* effector protein NleG6 mediates K27/K29-linked polyubiquitin chain modification, promoting autophagic degradation of the BRISC complex. This subsequently inhibits the NF-κB inflammatory pathway, thereby regulating tumor progression (Pan et al., 2025). Moreover, the gut bacterial metabolite guanidine can inhibit the ubiquitination and degradation of β-catenin by the E3 ubiquitin ligase Rnf128, thereby activating the Wnt signaling pathway and promoting the development of colorectal cancer (Table 7) (Lu et al., 2024).

## 4 Targeting microbial-ubiquitination/SUMOylation interactions for tumor therapeutic strategies

Altered ubiquitination and SUMOylation signaling are prevalent in various cancers and other pathological states. Targeting these pathways, particularly the ubiquitination pathway, is emerging as an attractive strategy in cancer therapy (Wertz and Wang, 2019). USP14 has been demonstrated to play a crucial role in cervical cancer as well as HPV + and HPV-HNSCC (Morgan et al., 2023). Its small-molecule inhibitor IU1 has been reported to exhibit antitumor activity in preclinical studies across multiple cancers, including cervical cancer (Xu et al., 2020; Srinivasan et al., 2023; Liu D. et al., 2024). Notably, another USP14 inhibitor, b-AP15, and its derivative VLX1570 have demonstrated anti-multiple myeloma potential in preclinical studies and advanced to Phase I clinical trials (NCT02372240). Despite the termination of the study due to severe pulmonary toxicity observed with VLX1570, the trial was unsuccessful. However, this exploration provides crucial evidence for the development of tumor therapies targeting deubiquitinating enzymes (Paulus et al., 2016; Rowinsky et al., 2020).

Given the prevalence of SUMOylation pathways across multiple cancers, significant progress has been made in developing inhibitors targeting this pathway, particularly SUMO E1 inhibitors. Among these, Subasumstat (TAK-981) is the most extensively studied and the only SUMOylation inhibitor to enter clinical trials (Kukkula et al., 2021). Beyond directly inhibiting tumor cell proliferation, TAK-981’s more significant role lies in its potent immunomodulatory function (Liu H. et al., 2025). Studies indicate that TAK-981 induces robust type I interferon (IFN-I) responses, promotes dendritic cell (DC) maturation, activates T cells and NK cells, and suppresses regulatory T cell (Treg) function. This transforms the immunosuppressive tumor microenvironment into an immune-activated one, enhancing the body’s antitumor immunity (Liu X. et al., 2025). Furthermore, Cambogin, a natural small molecule derived from the Garcinia plant, was found to effectively inhibit the interaction between the LANA protein and SUMO2. At low doses, it significantly suppressed the proliferation of KSHV-infected tumor cells and eliminated tumor growth in primary effusion lymphoma (PEL) in xenograft mouse models (Ding et al., 2019). Notably, Cambogin also exhibits significant antitumor activity against EBV-associated lymphomas, suggesting its potential as a broad-spectrum antiviral-antitumor candidate drug (Ding et al., 2019).

Interestingly, certain bacteria and fungi have been reported to exert antitumor effects through the ubiquitin-SUMO system. Metabolites such as propionate from gut bacteria (e.g., Bacteroides) induce HECTD2-mediated ubiquitination and degradation of EHMT2, activating TNFAIP1 to induce cancer cell apoptosis and inhibit colorectal cancer (Ryu et al., 2022). Probiotics like *Clostridium butyricum* inhibit CRC proliferation by enhancing MYC ubiquitin-proteasome degradation, sensitize to 5-FU chemotherapy, and improve response to anti-PD1 therapy, playing crucial roles in both chemotherapy and immunotherapy (Xu et al., 2023). Fungi of the genus Thelephora produce naphthalene derivatives, with xanthine being a representative example. These compounds interfere with ubiquitination and SUMOylation processes by inhibiting key proteases such as USP4/5 and SENP1, while also suppressing TNF-α, exhibiting anti-inflammatory and anti-cancer activities. They have become important candidate molecules for anti-cancer drug development (Bailly, 2022). Conversely, bioactive components in shiitake mushrooms suppress breast cancer progression by regulating the Nur77/HIF-1α signaling axis, particularly through promoting Nur77-dependent ubiquitin-proteasome degradation pathways (Zhang et al., 2020). These studies further expand our understanding of the link between microbial regulation of host ubiquitination systems and tumorigenesis, while also providing novel molecular targets and intervention strategies for the prevention and treatment of related cancers (Table 8).

**Table 8:** List of clinical therapeutic drugs targeting ubiquitination and tumor-suppressing microorganisms.

Inhibitors/Microorganisms	Target	Tumor type	Clinical trials	References
IU1	USP14	Cervical cancer	N/A	Xu et al. (2020), Srinivasan et al. (2023), and Liu D. et al. (2024)
VLX1570	USP14	Myeloma	NCT02372240	Paulus et al. (2016) and Rowinsky et al. (2020)
TAK-981	SUMO E1	Multiple myeloma	NCT04776018	Kukkula et al. (2021), Liu H. et al. (2025), and Liu X. et al. (2025)
		Lymphoma, non-Hodgkin	NCT04381650	
			NCT04074330	
		Head and neck cancer	NCT04065555	
		Hematologic neoplasms	NCT03648372	
Cambogin	SUMO2	Ebv-related lymphoma	N/A	Ding et al. (2019)
*Bacteroides*	TNFAIP1	Colorectal cancer	N/A	Ryu et al. (2022)
*Clostridium butyricum*	MYC	Colorectal cancer	N/A	Xu et al. (2023)
*Thelephora*	P-naphthylbenzene derivatives	Pancreatic cancer	N/A	Bailly (2022)
		Gastric cancer		

## 5 Summary and outlook

Microbial influence on tumors exhibits multidimensional characteristics: from dysbiosis of the normal microbiota, to the dynamic remodeling of the tumor microenvironment by tumor-associated microbes and their metabolites, to the intricate interactions between carcinogenic microbes and host cells and their components—all profoundly shape disease progression (Routy et al., 2024; Rahimi-Kolour et al., 2025; Pietropaolo et al., 2021). The ubiquitin and SUMOylation modification systems, as highly conserved reversible post-translational modification systems in eukaryotic cells, jointly maintain the dynamic equilibrium of the proteome. They ensure the orderly progression of core life activities such as DNA stability, gene expression regulation, cell cycle progression, and signal transduction. However, oncogenic microbes can ingeniously hijack this system, disrupting its homeostasis and promoting tumorigenesis by enhancing the stability and expression levels of key host proteins. For instance, high-risk HPV E6 specifically recruits USP46 to stabilize Cdt2/DTL via deubiquitination, thereby driving abnormal cell proliferation—a mechanism absent in low-risk HPV (Kiran et al., 2018). EBV latent membrane protein LMP1 directly interacts with glucose transporter GLUT1, inhibiting its K48-linked polyubiquitination and p62-mediated autophagy-lysosomal degradation pathway. This enhances GLUT1 stability, promoting the expansion of tumor-associated myeloid-derived suppressor cells (MDSCs) and exacerbating the immunosuppressive microenvironment in nasopharyngeal carcinoma (Cai et al., 2017). Such abnormal accumulation of key proteins induced by microbial interference holds potential as biomarkers for tumor diagnosis and prognosis.

**Figure 5:**
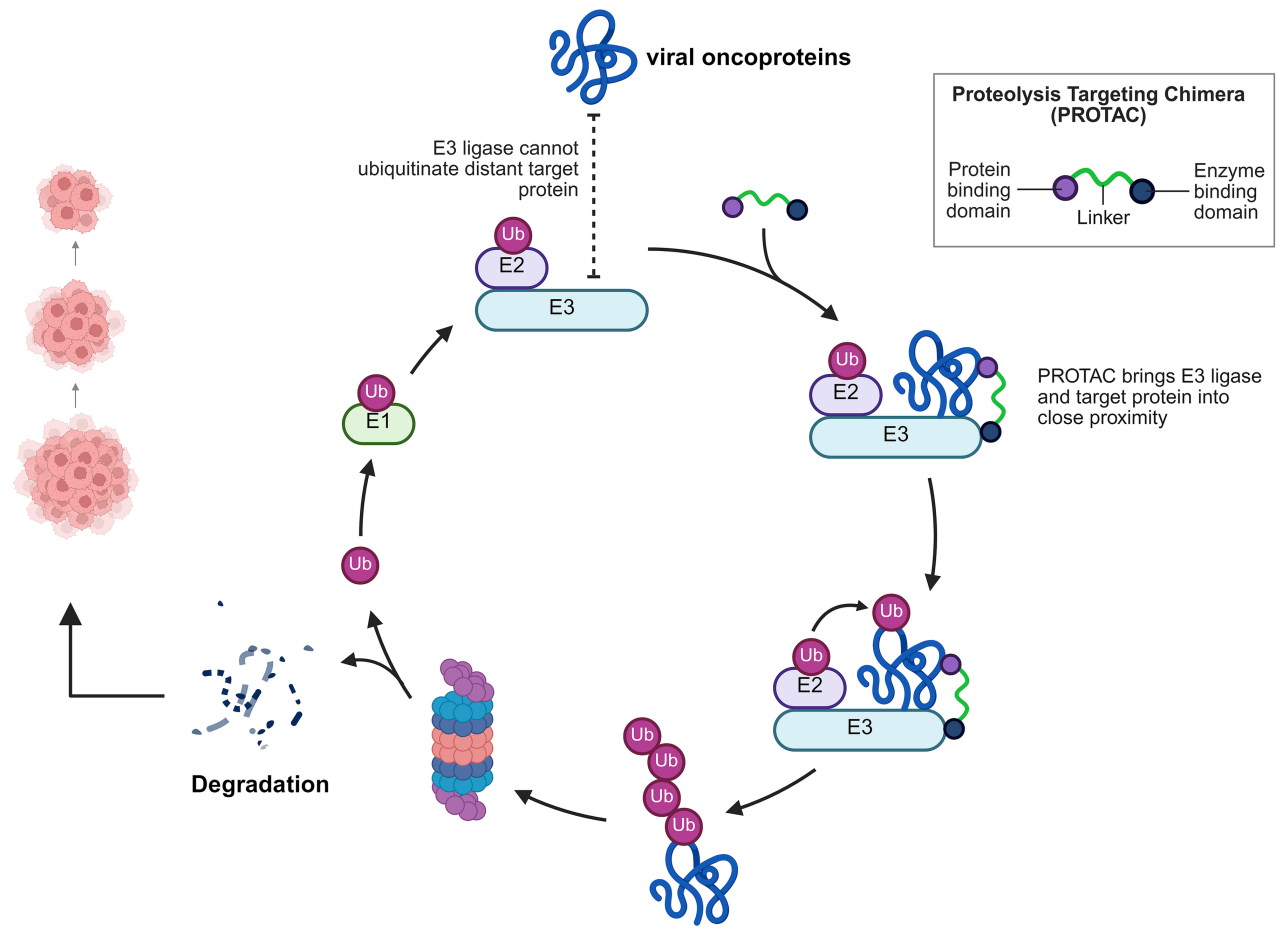
Protein-targeting chimeric antagonists (PROTACs) for the targeted degradation of viral oncogenes. Protein-targeting chimeric agents (PROTACs) based on the ubiquitin-proteasome system (UPS) can be employed to target viral oncoproteins modified by both ubiquitination and SUMOylation. These bifunctional molecules bind to the target protein at one end while recruiting E3 ubiquitin ligases at the other, thereby promoting the ubiquitination and subsequent degradation of viral oncoproteins (Ahmad et al., 2023).

Conversely, numerous oncogenic microbes can promote their own oncoprotein expression or evade degradation by modulating host ubiquitination and SUMOylation systems, leading to substantial accumulation within host cells and driving carcinogenesis. Since most of these microbial oncoproteins lack functional pockets suitable for small-molecule binding or primarily rely on extensive protein–protein interaction interfaces for function, they are often classified as “undruggable” targets. Protein degradation-targeting chimeras (PROTACs) offer a novel solution to this challenge (Xiong et al., 2022): these bifunctional molecules bind to any surface site on the target protein at one end while recruiting E3 ubiquitin ligases at the other, thereby selectively degrading the target protein via the host ubiquitin-proteasome pathway and completely eliminating its oncogenic activity (Figure 5). Currently, PROTAC strategies targeting influenza viruses, hepatitis viruses, SARS-CoV-2, and human cytomegalovirus proteins have demonstrated potential in the antiviral field (Liang et al., 2023), suggesting broad prospects for this approach in targeting microbial oncoproteins.

Meanwhile, advancements in nanomaterial technology are actively addressing the delivery challenges of PROTAC molecules. Stimulus-responsive nanocarriers enable precise controlled drug release based on tumor microenvironment characteristics—such as acidic pH, high glutathione concentrations, or specific enzyme expression—significantly enhancing therapeutic specificity while reducing systemic toxicity. For instance, the POLY-PROTAC nanosystem, activated by MMP-2 enzyme, has demonstrated tumor-site-specific drug release in experiments (Gao et al., 2022). Looking ahead, integrating nanomaterials with PROTAC technology holds promise for developing next-generation targeted degradation strategies. This approach enables precise intervention against microbial oncoproteins, offering novel pathways with clinical translation potential for treating related cancers.

## 6 Methods

### 6.1 Search strategy

#### 6.1.1 Databases

A comprehensive search was performed across six databases to cover both English and Chinese literature related to the research topic, including:

English databases: PubMed, Web of Science, Embase, Scopus.
Chinese database: China National Knowledge Infrastructure (CNKI).

These databases were selected for their extensive coverage of biomedical research, ensuring that studies on microbiology, protein post-translational modifications, and oncology were fully captured.

#### 6.1.2 Time range

Literature publication time was initially restricted to January 2020–October 2025, with the aim of prioritizing the acquisition of the latest research findings. Subsequently, to supplement the literature pool and incorporate classic studies that have laid the foundation for core mechanisms (e.g., pivotal research on microbial regulation of ubiquitination-related signaling pathways), the time axis was gradually expanded to earlier years (retrospectively extended to January 2010). This two-stage time range setting balances the timeliness and comprehensiveness of the review, ensuring that both the latest research advances and critical classic mechanisms in the field are fully covered.

#### 6.1.3 Search terms

A combination of MeSH terms (for English databases) and free-text words was used for retrieval to improve the comprehensiveness and accuracy of the search. The specific keyword combinations are shown in Table 9.

**Table 9:** Keywords searched developed according to JBI criteria.

JBI criteria	Keywords
Populations	Tumor, cancer, carcinoma, neoplasm
Concepts	Ubiquitination, ubiquitin, deubiquitinating enzyme, SUMOylation, SUMO, DeSUMOylating enzyme
Context	Microorganism, virus, bacterium, fungus, pathogen, therapeutic strategy, drug

### 6.2 Screening extraction

*Duplicate removal*: All retrieved literature records were imported into EndNote X21 software, and duplicate entries were automatically identified and removed. Manual checking was further performed to ensure no duplicates were missed.

*Title and abstract screening*: Two independent reviewers (Reviewer A and Reviewer B) screened the titles and abstracts of the remaining non-duplicate records. Literature that clearly did not meet the inclusion criteria (e.g., studies on non-tumor diseases, or not involving modification systems) was excluded.

*Full-text screening*: For literature that passed the title and abstract screening, full texts were retrieved. If full texts could not be obtained (e.g., unavailable through institutional databases), the corresponding authors were contacted via email. The two reviewers independently evaluated the full texts against the eligibility criteria. Disagreements during the screening process were resolved through discussion between the two reviewers; if consensus could not be reached, a third senior reviewer (with expertise in microbiology and oncology) was invited to arbitrate.
