# Supplementary figures and images for: Decoding microbial carcinogenic strategies: ubiquitination and SUMO modification
Source: Front Microbiol. 2025 Dec 19;16:1720153. doi: 10.3389/fmicb.2025.1720153 (PMC12757396; doi:10.3389/fmicb.2025.1720153)

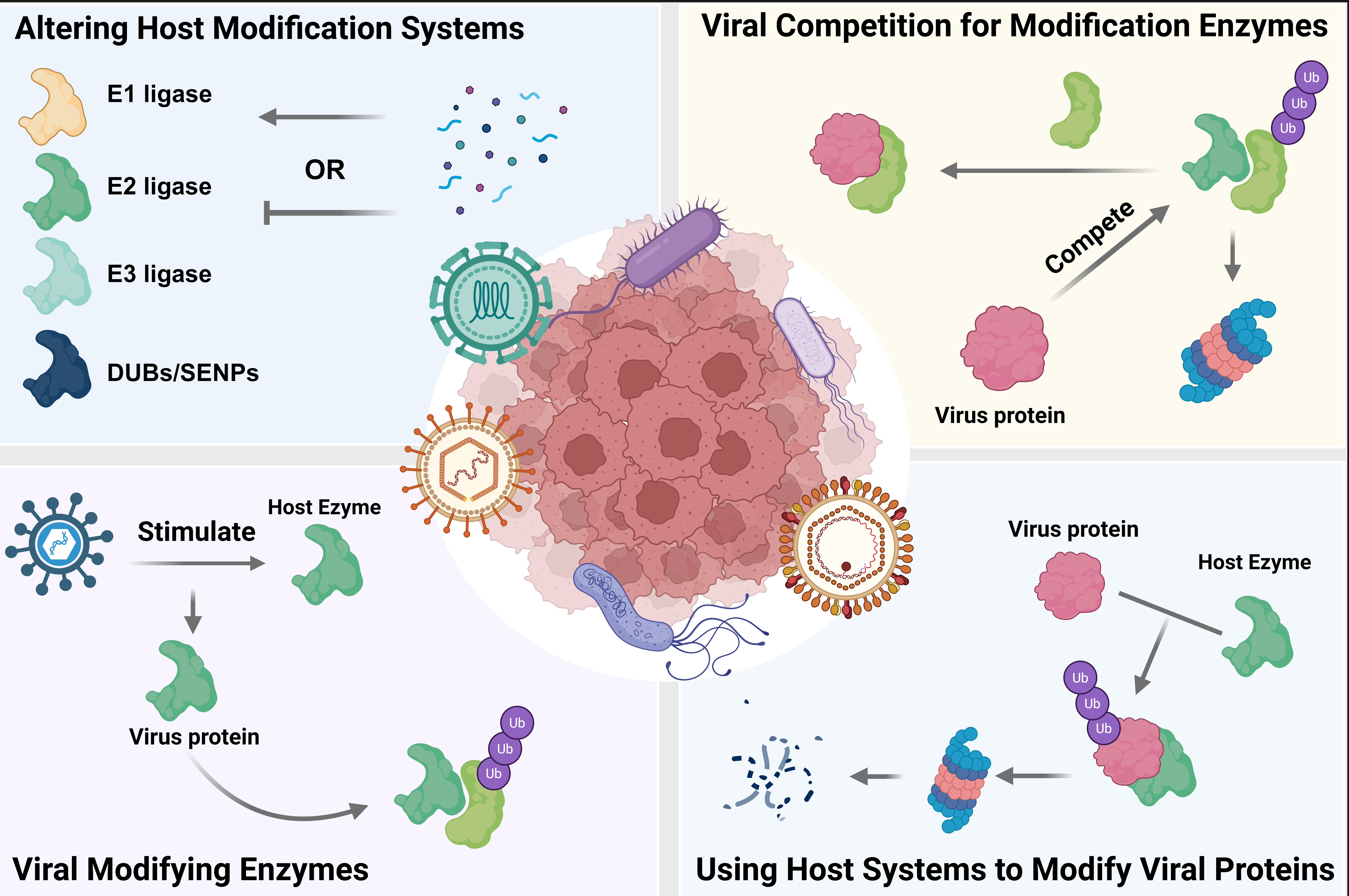

Supplement: Supplementary file 1 [file Image_1.jpeg]
